# Editorial: Towards Elimination of Dog Mediated Human Rabies

**DOI:** 10.3389/fvets.2017.00142

**Published:** 2017-08-31

**Authors:** Salome Dürr, Anna S. Fahrion, Lea Knopf, Louise H. Taylor

**Affiliations:** ^1^Veterinary Public Health Institute, University of Bern, Bern, Switzerland; ^2^Neglected Zoonotic Diseases, Department of Control of Neglected Tropical Diseases, World Health Organization, Geneva, Switzerland; ^3^Global Alliance for Rabies Control, Manhattan, KS, United States

**Keywords:** rabies, elimination, global health, resources, vaccination, dogs, one health, zoonosis

**Editorial on the Research Topic**

**Towards Elimination of Dog Mediated Human Rabies**

Rabies is a zoonotic viral disease with a high impact on human and animal health. The disease is almost 100% fatal after clinical signs appear and kills tens of thousands of people per year worldwide. About 99% of infections in humans are caused by rabid domestic dog bites. Human disease is related to poverty, with the highest burden in Asian and African low-income settings. Along with the group of neglected tropical diseases, increasingly recognized by high-level global health policy as indicators of functionality of health systems, rabies is scheduled for potential elimination as part of the UN sustainable development goals. In late 2015, the international rabies community, represented by more than 100 (mostly rabies endemic) countries, set a global target of eliminating human rabies mediated by dogs by 2030. Despite this momentum, rabies has received relatively less international attention compared with some of the other NTDs as of yet.

In this research topic, 123 authors contributed 15 articles (9 original research articles, 4 perspective pieces, and 2 reviews) from different regions in the world (4 from Australasia, 5 from Africa, 1 from Latin America, 4 global, and 1 theoretical) discussing various aspects of working towards the achievement of this goal. The collection brings together the experience and lessons learned from rabies control programs small and large, research aimed at improving the design and cost-effectiveness of rabies control programs, and analysis of the resources needed to expand rabies control efforts.

Our understanding of rabies control is sufficient, and the key tools are available to eliminate the disease. However, an overview by Fahrion et al. highlights the challenges and barriers to successfully *implementing* sustainable control of the disease. This article sets the scene for the whole collection, by discussing not just the gaps but also possible solutions for the socio-political, organizational, technical, and resource-linked issues that are being addressed by many different stakeholders. It highlights the need for applied research, a feature that has been taken up by most of the articles of this research topic.

Evidence that rabies can be locally eliminated has been built in recent years in a variety of settings, demonstrated here by Byrnes et al., Valenzuela et al., and Mpolya et al., describing successful state-wide programmes in Sikkim, India, Ilocos Norte, Philippines, and Southeast Tanzania and Pemba Island. All the programs were implemented by the government, in collaboration with one or more organizations, such as NGOs, WHO, OIE, national and international research institutes, and private foundations. Mass rabies vaccination in dogs and promotion of post-exposure prophylaxis (PEP) in humans were the common interventions, supported by dog population management (Byrnes et al.) and promotion of surveillance (Valenzuela et al.; Mpolya et al.), including the use of innovative methods such as mobile phone tools (Mpolya et al.), and the transition to intradermal PEP delivery (Mpolya et al.). Two facts were highlighted in all case studies, namely, the importance of a One Health approach demanding involvement of stakeholders from the veterinary and public health sectors and the challenge of sustaining progress in areas situated amidst rabies endemic areas.

From all projects there are lessons to be learned that can be used to support efforts elsewhere. This is particularly true for the coordinated approach to rabies control programs across Latin America and Caribbean (LAC), described by Del Rio Vilas et al., whose lessons derived are highly relevant to large-scale regional elimination goals. Although the target to eliminate rabies from LAC had to be reset four times since the implementation of the program in 1983 (it is currently set to 2022), a massive reduction of the human and animal rabies cases to (almost) 0 in most of the LAC countries has been achieved. One of the key messages is the need for adapted regional and national strategies to recognize that countries can vary enormously in their capacities.

Long-term intensive programs are required to achieve sustainable elimination and ensure that the reduction of cases is not followed by a resurgence of the incidence. The example from Morocco described by Darkaoui et al. demonstrates how inadequate implementation of the law, slack requests for responsibility from dog owners, and weak collaboration between Ministries impeded success in controlling rabies. Arief et al. also highlight the need for sustained control efforts in Bali, Indonesia, where dramatic reduction of rabies cases were achieved, but resurgence of disease has shown that elimination was still not possible.

What are the knowledge and action gaps that need to be addressed to implement sustainable rabies control programs at small and large scales? Concrete examples are addressed in articles of this research topic:

- The availability of high-quality surveillance data to support control efforts is absolutely vital. Unified reporting platforms have to be established (such as the epidemiological rabies bulletin for Africa proposed by Scott et al.) and sustainable community engagement has to be ensured for effective surveillance (Brookes et al.). The latter can only be achieved by the use of culturally adapted communication pathways.
- For concrete planning of control programs, detailed questions must be answered. For example, which subpopulation of dogs should be targeted in specific environments to reach the overall goal of elimination of rabies from the population? Theoretical modeling approaches can be used to answer such questions. In their setting, Leung and Davis identified the free-roaming owned dogs to be the most critical population to be vaccinated. Arief et al. conducted an observational study and identified puppies and dogs living in rural areas as having a higher risk of being unvaccinated, thus the focus should be set on these populations. Taylor et al. provide a comprehensive overview in their review on dog population management, an intervention for which evidence of its benefits with regards to rabies control is still lacking. The authors advocate for cost-effectiveness studies for dog population management and suggested that safe, effective, cheap, and injectable contraceptives for females should be a research priority to benefit management of dog populations.
- The important job of assessing the vaccination coverages achieved in free-roaming dogs is often neglected. Sambo et al. found that transect studies (counting vaccinated and unvaccinated dogs in the streets) soon after the campaign is cheap, quick, and provides good results. It is, therefore, more appropriate for routine monitoring of mass vaccination campaigns than household or school-based surveys.
- Not only in relation to rabies, but any control program, cost-effectiveness and identification of funding needs and options are crucial to ensure sustainability. Mindekem et al. found that a strategy that combines canine vaccination with the provision of PEP is more cost-effective in the long term than relying on PEP alone, particularly when communication across the human and veterinary health sectors is guaranteed to minimize unnecessary PEP application. Wallace et al. evaluated funding and capacity needs to reach the elimination of canine rabies globally and identified cheaper vaccine and increased efficiencies in vaccine delivery and application as ways to reduce these projected costs, but predicted that complementary dog population management interventions would markedly increase costs. Innovative financing mechanisms are needed to secure sufficient financial support, which is frequently a stumbling block to ensure that comprehensive vaccination plans can move forwards. Welburn et al. consider whether Development Impact Bonds could help to fill this funding gap. Such a finance model would enable investors taking on the risk of program delivery to ensure stricter management of implementation.

It is noteworthy that almost all contributions highlighted the need for an intersectoral approach involving all stakeholders, including the engagement of the communities. A One Health approach along the path to disease elimination (from resource allocation, to raising awareness, surveillance, implementation of control interventions, and eventual elimination) is a crucial requirement to realize the goal of 2030. To borrow words from Wallace et al., this research topic “hopes to stimulate and inform the necessary discussion on global and regional strategic planning, resource mobilization, and continuous execution of rabies virus elimination” that will be necessary to eliminate human-mediated canine rabies by 2030—a target that should not be reset.

## Author Contributions

All the authors were involved in writing the article and in editing contributions to this research topic. They all agree to the final version of this article.

## Conflict of Interest Statement

The authors declare that the research was conducted in the absence of any commercial or financial relationships that could be construed as a potential conflict of interest.

